# Supplementary material for: A joint modeling approach for longitudinal microbiome data improves ability to detect microbiome associations with disease
Source: PLoS Comput Biol. 2020 Dec 14;16(12):e1008473. doi: 10.1371/journal.pcbi.1008473 (PMC7769610; doi:10.1371/journal.pcbi.1008473)
Supplement: S1 Appendix — (PDF) [file pcbi.1008473.s002.pdf]

Microbiome Joint Model Tutorial

Pamela N. Luna

September 11, 2020

Contents

Introduction **1**

Pregnancy microbiome dataset **2**
Load data 2
Pre-processing 2
Formatting 3

Preliminary analyses **4**
Taxa abundances over time 4
Clustering microbiome samples 5
Verifying distribution fit 6
Removing low abundance taxa 8

Joint modeling analysis **8**
Longitudinal submodel 8
Event submodel 10
Joint model for longitudinal microbiome data 10

References **13**

Introduction

Here we provide a tutorial for how to apply our joint modeling methodology for longitudinal microbiome data [1] using the R programming language. To implement this methodology, we modified the existing `rstanarm` R package [2] to include functionality necessary for our model. More specifically, we updated the joint modeling implementation [3] by:

- Adding functionality for an offset term in the longitudinal submodel
- Parameterizing the event submodel to include relative abundances rather than the predicted counts from the longitudinal submodel
- Introducing a scaling parameter to modify the scale of the relative abundances in the event submodel

These changes are currently available via the development version of `rstanarm` and can be installed in R using the following commands.

```r
if (!require("remotes")) {
  install.packages("remotes")
}
remotes::install_github("stan-dev/rstanarm", INSTALL_opts = "--no-multiarch")
```

Additional instructions for installing this software are available on the rstanarm GitHub page. The documentation for the rstanarm software is available via CRAN or can be built using the above command by including the build_vignettes = TRUE argument (note that this significantly increases installation time).

Pregnancy microbiome dataset

We use a pregnancy microbiome dataset to illustrate how to perform joint modeling on longitudinal microbiome and time-to-event data. The dataset is derived from a case-control study by DiGiulio et al. examining the microbial compositions of various body sites of women during pregnancy [4]. We use a pre-formatted version of this dataset from a paper by Zhang, et al. on longitudinal modeling of microbiome data [5]. In this tutorial we provide the basic steps necessary to analyze how changes in specific taxon abundances in the vaginal microbiome during pregnancy affect the time-to-delivery.

Load data

The dataset for this tutorial can be downloaded directly from GitHub using the readr R package [6].

```
library(readr)
url <- paste0(
  "https://raw.githubusercontent.com/abbyyan3/NBZIMM-tutorial/",
  "master/NBMM-longitudinal-temporal-data/"
)
dat.samp.tab <- read.csv(
  url(paste0(url, "temporal%20spatial%20clinical%20merged.csv")),
  row.names = 1
)
dat.otu.tab <- read.csv(
  url(paste0(url, "temporal%20spatial%20otu.csv")),
  row.names = 1
)
dat.tax.tab <- read.csv(
  url(paste0(url, "temporal%20spatial%20taxonomy.csv")),
  row.names = 1
)
```

For this analysis we retrieve three data tables:

1. dat.samp.tab - metadata table containing information for each sample
2. dat.otu.tab - OTU table with abundances for each sample
3. dat.tax.tab - taxonomy table for the taxa observed in the data

We use these tables to construct a phyloseq object using the phyloseq R package [7]. In addition to allowing the user to upload data tables directly, the package provides functionality for importing data using common microbiome data formats. Tutorials for importing data and performing exploratory analyses using phyloseq can be found at http://joey711.github.io/phyloseq/.

Pre-processing

In general, the pre-processing necessary for analysis will be highly dependent on the dataset and personal preferences. The taxonomy table for this dataset contains entries with special characters that cannot be used to label rows and columns, so we remove those characters from the data.

```
dat.tax.tab <- apply(dat.tax.tab, 2, function(x) gsub("\\[|\\]", "", x))
dat.tax.tab <- apply(dat.tax.tab, 2, function(x) gsub(":|-", "_", x))
```

The row names of the taxonomy table and the column names of the OTU table must be identical for importing the data into `phyloseq`. The column names in the OTU table were prefixed with an 'X' when loaded to create valid data frame column names. To have matching and valid row and column names between these two tables, we introduce a new 'OTU_' prefix for the OTU labels.

```
otu.names <- paste0("OTU_", rownames(dat.tax.tab))
rownames(dat.tax.tab) <- otu.names
colnames(dat.otu.tab) <- otu.names
```

To import the data tables to a `phyloseq` object, we create the respective objects for the metadata (or sample data), OTU, and taxonomy tables.

```
library(phyloseq)
SAM <- sample_data(dat.samp.tab)
OTU <- otu_table(dat.otu.tab, taxa_are_rows = FALSE)
TAX <- tax_table(as.matrix(dat.tax.tab))
```

We then import the data to a `phyloseq` object and perform a few basic operations. The `tax_glom` function combines the samples at a given classification level – we will analyze data at the genus level. The `subset_samples` function is used to select only the vaginal samples we will use for analysis and to remove samples collected postpartum. We have also loaded the `tidyverse` R package, a collection of tools such as `ggplot2` for plotting and `dplyr` for data formatting that improve the efficiency of data analysis [8].

```
library(tidyverse)
ps.genus <- phyloseq(OTU, TAX, SAM) %>%
  tax_glom(taxrank = "Genus") %>% # Combine by genus
  subset_samples(BodySite == "Vaginal_Swab") %>% # Look only at vaginal samples
  subset_samples(GDColl <= GDDel) # Remove samples collected after delivery
```

We relabel the taxa using the genus names now that the OTU abundances have been combined at the genus level. If you choose not to relabel the taxa names manually, the `phyloseq` software will label the combined OTUs based on the OTU with the largest abundance within the genus.

```
taxa_names(ps.genus) <- make.names(tax_table(ps.genus)[, "Genus"], unique = TRUE)
```

Formatting

Formatting metadata prior to exploratory analyses can be helpful, but determining the optimal formatting for your model might be an iterative process. We remove samples that were collected prior to $t = 0$, the beginning of gestation, and create and simplify variables necessary for the longitudinal and event submodels. Additionally, we adjust the time scale of our data to weeks rather than days or months. Data with a high resolution (e.g., gestational days) will have less drastic changes per unit of time. Conversely, a low resolution time scale (e.g., gestational months) could have time units with multiple samples which could reduce the model's ability to detect changes over time.

```
metadata <- data.frame(sample_data(ps.genus)) %>%
  filter(GDColl >= 0) %>% # Remove samples prior to pregnancy
  mutate(GWColl = GDColl / 7) %>% # Transform time scale to weeks
  mutate(GWDel = GDDel / 7) %>% # Transform time scale to weeks
  mutate(Preterm = (Outcome != "Term")) %>%
  mutate(high_income = SES_household_income %in%
    c(
      "$60,000 - $80,000", "$80,000 - $100,000", "$100,000 - $150,000",
```

```r
    "$150,000 - $200,000", "$250,000 or more"
  )) %>%
  mutate(non_hispanic_white = (Ethnicity == "Non-hispanic" &
    Race == "White")) %>%
  mutate(Delivery = 1) %>%
  mutate(History_of_preterm_delivery = factor(History_of_preterm_delivery)) %>%
  mutate(TrimColl = factor(TrimColl))
```

Next we create a longitudinal data frame by combining the metadata and taxa data for each longitudinal microbiome sample. Note that the metadata in this example contains total counts (library sizes) for our taxa data. If not included in your dataset, this variable is calculated by taking the sum of the taxa abundances across each sample and should be added to the longitudinal data frame for use in the longitudinal model prior to the removal of any taxa.

```r
taxa.data <- data.frame(otu_table(ps.genus))
long.data <- metadata %>%
  left_join(taxa.data %>% rownames_to_column("SampleID"), by = "SampleID")
```

Preliminary analyses

Our methodology works best as a tool to verify and quantify relationships identified via preliminary analyses. In this section we discuss basic methods for determining taxa that warrant further examination. Note that this is only a small collection of simple but useful analyses – more advanced analyses could provide additional insights.

Taxa abundances over time

In this example we look at the ten most abundant taxa sorted by mean relative abundance over time. Note that it could be informative to explore the taxa data sorted by other summary statistics such as sum or variance.

```r
genus.names <- taxa_names(ps.genus)
taxa.means <- apply(long.data[, genus.names] / long.data$LibrarySize, 2, mean)
head(sort(taxa.means, decreasing = TRUE), 10)
```

```
## Lactobacillus    Prevotella   Gardnerella    Finegoldia   Megasphaera
##   0.812084781   0.059514010   0.034658527   0.011409867   0.009883797
##     Atopobium     Dialister    Ureaplasma  Anaerococcus Peptoniphilus
##   0.009376003   0.008647069   0.006081102   0.004270534   0.003983642
```

We compare the trajectories for the top 10 genera over time using the time related outcome of preterm birth, which is defined as a time-to-delivery < 37 weeks.

```r
top.taxa <- names(head(sort(taxa.means, decreasing = TRUE), 10))
top.data <- long.data %>%
  select(GWColl, Preterm, all_of(top.taxa), LibrarySize) %>%
  mutate_at(top.taxa, ~ . / LibrarySize) %>% # transform to relative abundances
  gather(key = "genus", value = "abundance", all_of(top.taxa), factor_key = TRUE)

ggplot(data = top.data, aes(x = GWColl, y = abundance, color = genus)) +
  geom_smooth(se = FALSE) +
  xlab("Gestational weeks") +
  ylab("Relative abundance") +
```

```
  scale_color_brewer("Genus", palette = "Paired") +
  facet_wrap(~Preterm)
```

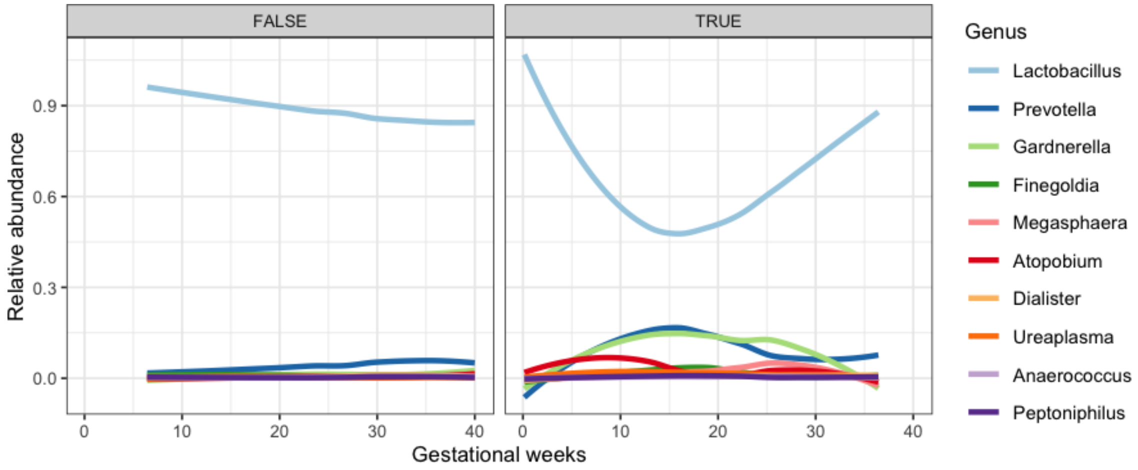

This plot shows consistently higher abundances of *Lactobacillus* in term versus preterm outcomes. Additionally, abundances of *Prevotella* and *Gardnerella* increase more with preterm births. These results indicate that the three most abundant taxa in our dataset are worth further exploration. However, these results do not rule out other lower abundance taxa that might be relevant.

Clustering microbiome samples

Clustering microbiome samples can reveal common microbial composition patterns and illustrate how those patterns might relate to outcomes. There are many approaches for clustering microbiome data that are outside the scope of this tutorial but should be further researched prior to analyzing a microbiome data set. For this example we perform partitioning around medoids (PAM) clustering on the Bray-Curtis dissimilarity matrix for all samples using four clusters.

```
ld.norm <- long.data %>%
  mutate_at(genus.names, ~ . / LibrarySize) # transform to relative abundances

bc.diss <- vegan::vegdist(ld.norm %>% select(all_of(genus.names)), method = "bray")
p.clust <- cluster::pam(bc.diss, diss = TRUE, k = 4, cluster.only = TRUE)
```

We then plot a heatmap of the five most abundant genera with samples grouped by cluster.

```
hm.data <- ld.norm %>%
  mutate(Cluster = factor(p.clust)) %>%
  mutate(Preterm = factor(Preterm, levels = c(TRUE, FALSE), labels = c("Yes", "No"))) %>%
  arrange(Cluster, Preterm) %>%
  column_to_rownames("SampleID")

anno.colors <- list(
  Preterm = c("Yes" = "#cc4ee0", "No" = "#e89829"),
  Cluster = c("1" = "#A6CEE3", "2" = "#1F78B4", "3" = "#B2DF8A", "4" = "#33A02C")
)

pheatmap::pheatmap(t(hm.data %>% select(all_of(top.taxa[1:5]))),
  cluster_cols = FALSE,
  cluster_rows = FALSE,
  annotation_col = hm.data %>% select(Preterm, Cluster),
```

```
  show_colnames = FALSE,
  annotation_colors = anno.colors
)
```

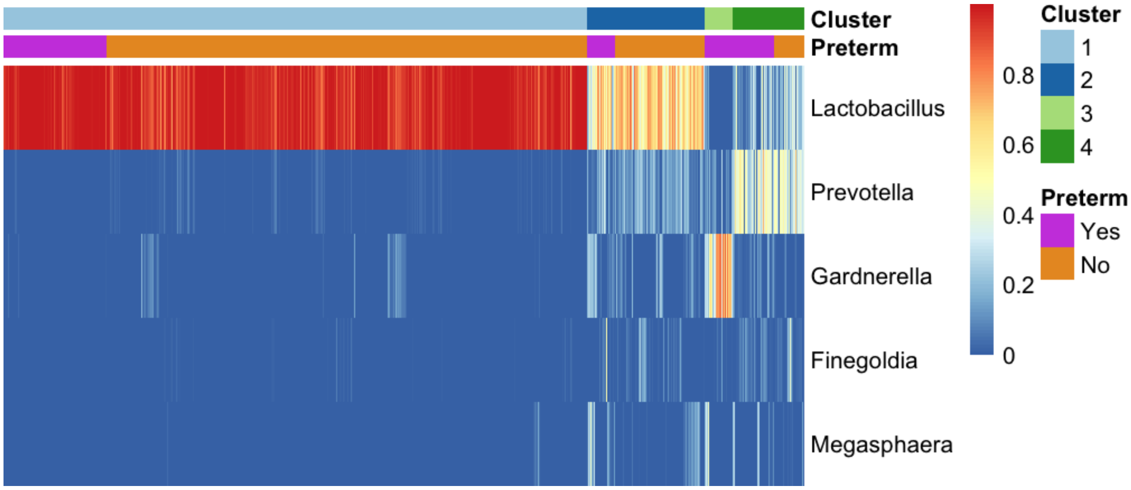

Cluster membership for the samples is shown along the top bar, and the preterm outcome is shown along the second bar with pink indicating samples from women who delivered preterm. Clusters 1 and 2 contain the majority of samples, which are dominated by high abundances of *Lactobacillus*, and do not have a large proportion of preterm outcomes (18% and 23%, respectively). Cluster 3 is a small group that consists of *Gardnerella*-dominant samples, all of which are associated with preterm outcomes. Cluster 4 contains *Prevotella*-dominant samples with 59% of the samples having a preterm outcome. These clustering results further support examining the three most abundant taxa and indicate that increased abundances of *Gardnerella* and *Prevotella* might be positively associated with preterm outcomes.

Verifying distribution fit

The longitudinal submodel assumes that the taxon follows a negative binomial distribution. The negative binomial distribution is a discrete probability distribution that is well-suited for non-negative and overdispersed microbiome count data. However, a negative binomial distribution will not be an optimal representation of taxa that exhibit bimodality or zero-inflation, which could impact the accuracy of joint modeling results. To assess how well a negative binomial distribution represents taxon abundances, we plot a histogram of the taxon abundances and overlay a plot of the density of counts sampled from a negative binomial distribution with the sample mean and dispersion observed in our data.

As an example of how to verify the distribution of taxa within the dataset, we will examine the two most abundant taxa, *Lactobacillus* and *Prevotella*.

Lactobacillus

We first calculate the sample parameters from the observed data for *Lactobacillus*. The sample dispersion is defined as $\theta = \frac{\mu^2}{\sigma^2 - \mu}$, where μ is the sample mean and σ^2 is the sample variance.

```
samp.mean <- mean(taxa.data$Lactobacillus)
samp.var <- var(taxa.data$Lactobacillus)
samp.disp <- samp.mean^2 / (samp.var - samp.mean)
```

Using these parameter estimates, we draw 1000 samples from a negative binomial distribution. (Note that the dispersion parameter θ can also be referred to as the size or shape parameter.) We overlay a density plot of these samples onto a histogram of the observed values.

```r
nb.dist <- data.frame(samp = rnbinom(1000, size = samp.disp, mu = samp.mean))
ggplot(taxa.data, aes(x = Lactobacillus)) +
  geom_histogram(aes(y = ..density..), # Use density instead of counts
    color = "black", fill = "white"
  ) +
  geom_density(data = nb.dist, aes(samp), alpha = .2, fill = "red")
```

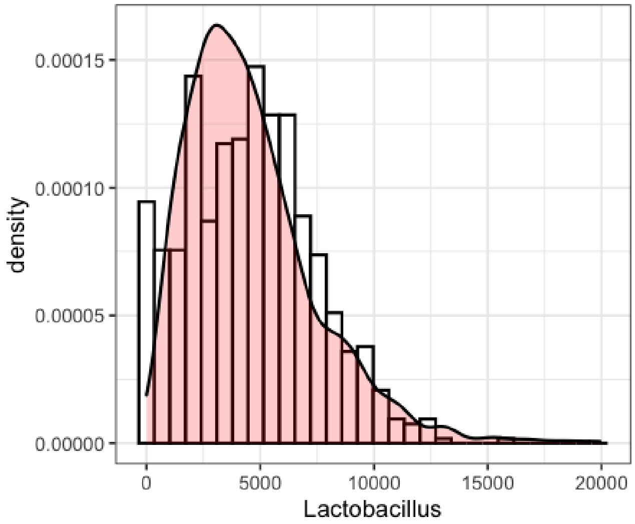

Looking at this plot, we see more zero abundance *Lactobacillus* samples in our data than expected for a negative binomial distribution with the observed sample mean and sample dispersion. Because the distribution of *Lactobacillus* does not closely align with the expected density, the longitudinal submodel would not provide a good fit for the data.

Prevotella

We construct the same plot using *Prevotella* abundances.

```r
samp.mean <- mean(taxa.data$Prevotella)
samp.var <- var(taxa.data$Prevotella)
samp.disp <- samp.mean^2 / (samp.var - samp.mean)
nb.dist <- data.frame(samp = rnbinom(1000, size = samp.disp, mu = samp.mean))
ggplot(taxa.data, aes(x = Prevotella)) +
  geom_histogram(aes(y = ..density..), # Use density instead of counts
    color = "black", fill = "white"
  ) +
  geom_density(data = nb.dist, aes(samp), alpha = .2, fill = "red")
```

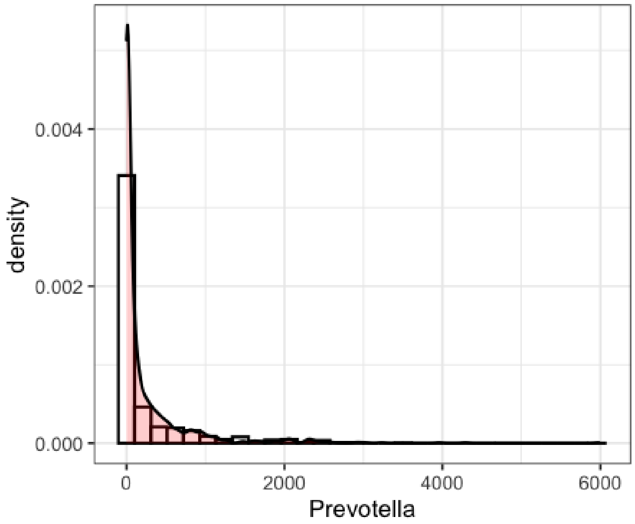

The observed abundances of *Prevotella* correspond with the expected density of a negative binomial distribution with the observed sample mean and dispersion, indicating that *Prevotella* would work well with this approach.

Removing low abundance taxa

The previous analyses highlight highly abundant taxa that are typically of interest in microbiome data analysis. Some researchers might be interested in analyzing a large number of taxa or low abundance taxa with a detectable effect on the time-to-event outcome. In these instances, we recommend filtering out very low abundance taxa that would be insufficient for joint modeling analysis. A few options would be to exclude taxa that:

- have a mean/median/sum below a specified threshold
- are zero in a given number of samples (e.g., taxon is zero in more than 10% of samples)
- are below a threshold in a given number of samples (e.g., taxon is below 1% abundance in more than 10% of samples)

Filtering taxa is relatively easy using the `prune_taxa()` function in the `phyloseq` R package. However, note that calculation of the total counts (library sizes) must be performed prior to filtering taxa.

Joint modeling analysis

In this section we outline the steps to perform joint modeling on longitudinal microbiome data with time-to-event outcomes using the formatted data. To construct this model we use the `rstanarm` R package, which simplifies the process of analyzing Bayesian regression models in R via Stan.

```
library(rstanarm)
```

Longitudinal submodel

The longitudinal submodel is a negative binomial mixed effects model and serves as a predictive model for the relative abundances included in the event component of the joint model. The fixed effects included in the model should be methodically chosen using variable selection and/or exploratory analyses. For a longitudinal model the random effects are typically represented with a subject-specific random intercept and a time covariate for the random slope. Although the event submodel will use the gestational weeks variable `GWColl`, the longitudinal submodel for *Prevotella* is more accurate when using the trimester variable `TrimColl` for the

random slope. Finally, we include an offset term for the log library size of each sample to adjust for variation of total counts across samples.

```
long.model <- Prevotella ~ GWColl + History_of_preterm_delivery +
  Preeclampsia + non_hispanic_white +
  (TrimColl | SubjectID) + # random effects
  offset(log(LibrarySize)) # offset term

long.data <- long.data %>%
  select(
    Prevotella, GWColl, History_of_preterm_delivery, Preeclampsia,
    non_hispanic_white, TrimColl, SubjectID, LibrarySize
  )
```

Because the model predictions from the longitudinal submodel are incorporated into the event submodel, it is especially important to test the predictive fit of the longitudinal model. For testing purposes, the model can be built using external software, such as the `BhGLM` [9] or `brms` [10] R packages, or using the `stan_glmer` or `stan_mvmer` included in `rstanarm`. We use `stan_mvmer` here, which is the function used within the joint modeling function.

```
mvmer.fit <- stan_mvmer(
  formula = long.model, data = long.data, family = neg_binomial_2,
  seed = 54321, iter = 3000, chains = 1
)
summary(mvmer.fit)
```

Note that it is preferable to use multiple chains but we use only one here for simplicity. Longitudinal model results can be viewed using the `summary` function, the output of which we exclude here for brevity.

The longitudinal model predictions do not require high accuracy but should have a relatively good fit. We plot the predicted relative abundances against the observed relative abundances and find that we have sufficient model predictions.

```
pp <- posterior_predict(mvmer.fit)
pp.means <- apply(pp, 2, mean)
qplot(
  x = Prevotella / LibrarySize, y = pp.means / LibrarySize, data = long.data,
  xlim = c(0, 1), ylim = c(0, 1), xlab = "Observed values", ylab = "Predicted values"
) +
  geom_abline(slope = 1, intercept = 0, color = "red")
```

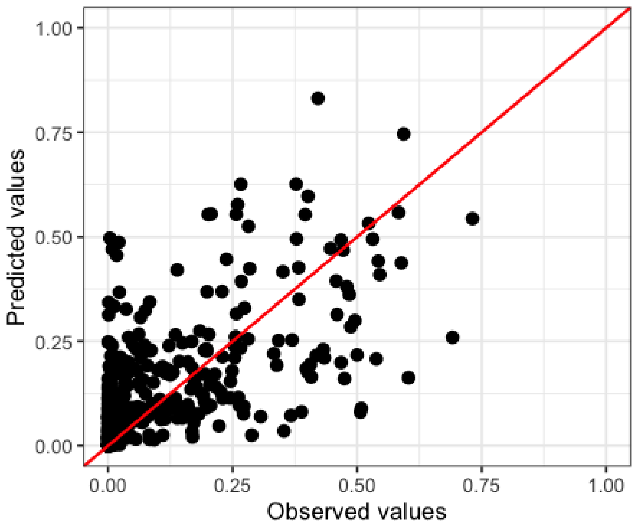

Event submodel

The event submodel is the foundation of the joint model into which the longitudinal model values will be introduced. In this example we model the time-to-delivery `GWDel` outcome. Variable selection for the event submodel should be performed using standard methods for Cox proportional hazards modeling. Specifically, users should verify that covariates included in the model do not violate the proportional hazards assumption using a function such as `cox.zph` in the `survival` R package [11].

```
event.model <- Surv(GWDel, Delivery) ~ Preeclampsia + non_hispanic_white + high_income

event.data <- metadata %>%
  select(SubjectID, GWDel, Delivery, Preeclampsia, non_hispanic_white, high_income) %>%
  distinct()
```

The event data frame should have one row for each subject. We select the event submodel covariates from the metadata and remove duplicates to obtain the necessary data frame.

Joint model for longitudinal microbiome data

Performing joint model analysis is relatively straightforward once the longitudinal and event submodels are defined. Aside from the `scale_assoc` parameter we have introduced, arguments for the `stan_jm` function are detailed in the `rstanarm` documentation. However, we will highlight important recommendations and requirements for some arguments based on our methodology.

- **Scaling parameter:** Our update to the joint modeling function in `rstanarm` introduces the `scale_assoc` argument, which represents the scaling parameter ϕ discussed in detail in our manuscript. This parameter multiplicatively scales the predicted relative abundances included in the event submodel. Although the optimal value for this parameter might vary based on the data and specific taxon you are modeling, `scale_assoc=10` is often sufficient for detecting the taxon abundance effect size and maintaining an interpretable result.
- **Data-dependent arguments:** These arguments are specific to the dataset and user-defined variables.
  - `formulaLong`, `dataLong` - formula and data frame for the longitudinal submodel
  - `formulaEvent`, `dataEvent` - formula and data frame for the event submodel
  - `time_var` - the time variable for the longitudinal data which must be included as a fixed effect in the longitudinal model and must be on the same time scale as the time-to-event outcome
  - `id_var` - the variable that identifies subjects which must be included in both longitudinal and event data
- **Model required arguments:** To perform joint modeling with longitudinal microbiome data as presented in our manuscript, these argument settings are required.
  - `family = neg_binomial_2` - defines the distribution for the longitudinal submodel
  - `assoc = 'muvalue'` - defines how longitudinal model values are parameterized in the joint model
- **Bayesian modeling parameters:**
  - `adapt_delta` and `max_treedepth` - These are tuning parameters specific to the MCMC implementation used in `rstanarm`. The software provides warning messages when these values should be modified from their default values.
  - `init_r` - This argument sets the magnitude of the range for the initial parameter values, which will be uniformly sampled from the range [-`init_r`,`init_r`]. The default value of `init_r=2` can result in initialization values that are too large for the event model parameters and cause the model building to fail. We recommend setting this argument to a smaller value, such as `init_r=0.5`, for this modeling approach.

We use the following command to construct the joint model as outlined above for our example pregnancy microbiome dataset.

```r
jm.fit <- stan_jm(
  formulaLong = long.model, dataLong = long.data,
  formulaEvent = event.model, dataEvent = event.data,
  time_var = "GWColl", id_var = "SubjectID",
  family = neg_binomial_2, assoc = "muvalue",
  scale_assoc = 10,
  adapt_delta = 0.9, max_treedepth = 15, init_r = 0.5,
  seed = 54321
)
```

The print function shows a short summary of the posterior distributions for the estimated parameters. A more detailed summary of the model results can be printed using the summary function (not shown here for brevity).

```r
print(jm.fit)
```

```
## stan_jm
##  formula (Long1): Prevotella ~ GWColl + History_of_preterm_delivery + Preeclampsia +
##     non_hispanic_white + (TrimColl | SubjectID) + offset(log(LibrarySize))
##  family  (Long1): neg_binomial_2 [log]
##  formula (Event): Surv(GWDel, Delivery) ~ Preeclampsia + non_hispanic_white + high_income
##  baseline hazard: bs
##  assoc:           muvalue (Long1)
## ------
##
## Longitudinal submodel: Prevotella
##                              Median MAD_SD exp(Median)
## (Intercept)                  -5.618  0.819  0.004
## GWColl                        0.032  0.017  1.033
## History_of_preterm_delivery1  1.093  0.905  2.983
## PreeclampsiaTRUE              2.132  0.988  8.432
## non_hispanic_whiteTRUE       -1.331  0.941  0.264
## reciprocal_dispersion         0.370  0.020     NA
##
## Event submodel:
##                        Median  MAD_SD  exp(Median)
## (Intercept)             -4.107   0.273   0.016
## PreeclampsiaTRUE         0.969   0.543   2.634
## non_hispanic_whiteTRUE   0.044   0.418   1.045
## high_incomeTRUE         -0.003   0.367   0.997
## Long1|muvalue            0.403   0.204   1.496
## b-splines-coef1        -21.677  18.437      NA
## b-splines-coef2         -2.812   8.812      NA
## b-splines-coef3         -6.345   2.552      NA
## b-splines-coef4          2.929   0.472      NA
## b-splines-coef5          5.612   0.802      NA
## b-splines-coef6          4.615   1.240      NA
##
## Group-level error terms:
##  Groups    Name              Std.Dev. Corr
##  SubjectID Long1|(Intercept) 2.758
##            Long1|TrimColl2   1.742     0.04
##            Long1|TrimColl3   2.635    -0.40  0.54
## Num. levels: SubjectID 40
```

```
##
## Sample avg. posterior predictive distribution
## of longitudinal outcomes:
##                  Median  MAD_SD
## Long1|mean_PPD 357.260  78.536
##
## ------
## For info on the priors used see help('prior_summary.stanreg').
```

These results show that the posterior median for the taxon abundance effect size (listed in the results as `Long1|muvalue` in the event submodel) is $\hat{\alpha} = 0.403$. The hazard ratio for this parameter can be calculated as $\exp(0.403) = 1.5$. Because we have scaled our relative abundances by 10, a unit increase for this model is equivalent to a 10% increase in relative abundance. Therefore, the predicted hazard ratio indicates that for a 10% increase in *Prevotella* abundance the hazard of delivery increases 1.5-fold.

We would like to note that in the manuscript, we use the posterior mean as the predictor for the taxon abundance effect size. Both the posterior mean and median are commonly used as parameter point estimates in Bayesian modeling. Ideally, as is the case with this example, the posterior mean and median will be approximately equal.

We plot the posterior distributions for model parameters using the `bayesplot` package [12].

```
library(bayesplot)
posterior <- as.matrix(jm.fit)
mcmc_areas(posterior,
  pars = c(
    "Assoc|Long1|muvalue",
    "Event|PreeclampsiaTRUE", "Event|non_hispanic_whiteTRUE", "Event|high_incomeTRUE"
  ),
  prob = 0.8
) +
  ggtitle("Joint model posterior distributions", "with medians and 80% intervals")
```

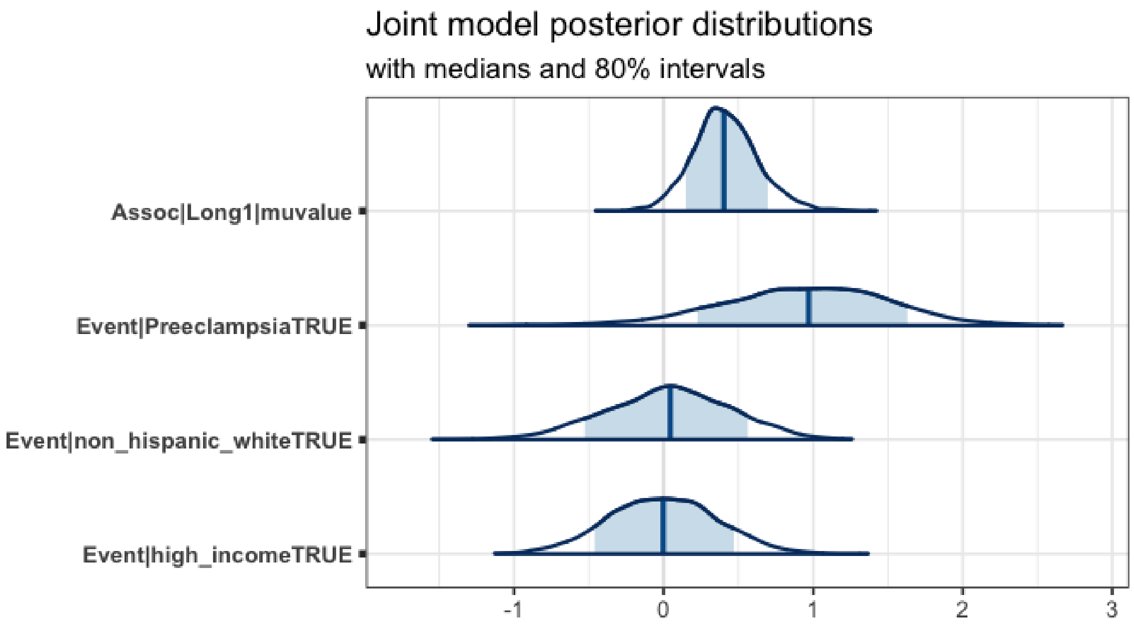

Model results can be further analyzed by examining posterior predictions for the longitudinal and event submodels. Details for performing these predictive analyses are thoroughly documented in the `rstanarm` joint modeling vignette.

References

1. Luna PN, Mansbach JM, Shaw CA. A joint modeling approach for longitudinal microbiome data improves ability to detect microbiome associations with disease. PLOS Computational Biology. 2020.

2. Goodrich B, Gabry J, Ali I, Brilleman S. Rstanarm: Bayesian applied regression modeling via Stan. 2020. Available: http://mc-stan.org/

3. Brilleman S, Crowther M, Moreno-Betancur M, Buros Novik J, Wolfe R. Joint longitudinal and time-to-event models via Stan. Proceedings of StanCon 2018. 2018. Available: https://github.com/stan-dev/stancon_talks/

4. DiGiulio DB, Callahan BJ, McMurdie PJ, Costello EK, Lyell DJ, Robaczewska A, et al. Temporal and spatial variation of the human microbiota during pregnancy. Proceedings of the National Academy of Sciences of the United States of America. 2015;112: 11060–5. doi:10.1073/pnas.1502875112

5. Zhang X, Pei Y-F, Zhang L, Guo B, Pendegraft AH, Zhuang W, et al. Negative Binomial Mixed Models for Analyzing Longitudinal Microbiome Data. Frontiers in Microbiology. 2018;9: 1683. doi:10.3389/fmicb.2018.01683

6. Wickham H, Hester J, Francois R. Readr: Read rectangular text data. 2018. Available: https://CRAN.R-project.org/package=readr

7. McMurdie PJ, Holmes S. Phyloseq: An r package for reproducible interactive analysis and graphics of microbiome census data. PLoS ONE. 2013;8: e61217. Available: http://dx.plos.org/10.1371/journal.pone.0061217

8. Wickham H, Averick M, Bryan J, Chang W, McGowan LD, François R, et al. Welcome to the tidyverse. Journal of Open Source Software. 2019;4: 1686. doi:10.21105/joss.01686

9. Yi N. BhGLM: Bayesian hierarchical glms and survival models, with applications to genomics and epidemiology. 2019. Available: https://github.com/nyiuab/BhGLM

10. Bürkner P-C. Advanced Bayesian multilevel modeling with the R package brms. The R Journal. 2018;10: 395–411. doi:10.32614/RJ-2018-017

11. Therneau TM. A package for survival analysis in s. 2015. Available: https://CRAN.R-project.org/package=survival

12. Gabry J, Mahr T. Bayesplot: Plotting for bayesian models. 2020. Available: https://mc-stan.org/bayesplot
