# Supplementary figures and images for: A joint modeling approach for longitudinal microbiome data improves ability to detect microbiome associations with disease
Source: PLoS Comput Biol. 2020 Dec 14;16(12):e1008473. doi: 10.1371/journal.pcbi.1008473 (PMC7769610; doi:10.1371/journal.pcbi.1008473)

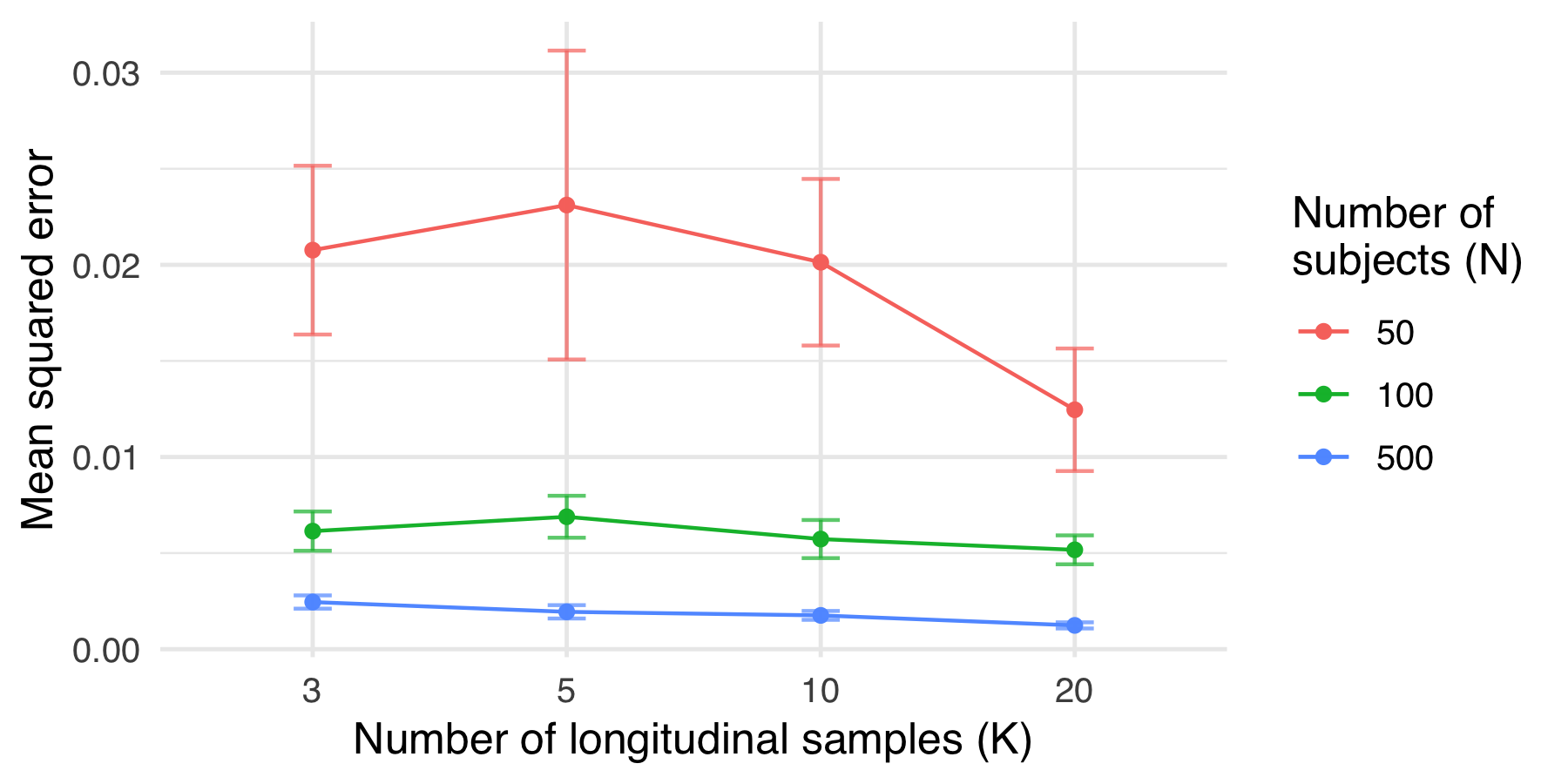

Supplement: S1 Fig — Application of the joint modeling methodology on simulated data sets with varying sizes for the number of subjects (N) and number of longitudinal samples (K) shows that the model retains accuracy with smaller sample sizes. The mean squared errors (MSEs) for the effect size predictions remain low with sample sizes as small as N = 100 with any number of longitudinal samples. The MSEs for effect size predictions are larger with sample size N = 50, but the MSEs are reduced with an increased number of longitudinal samples K. (TIF) [file pcbi.1008473.s003.tif]
