# Supplementary material for: A joint modeling approach for longitudinal microbiome data improves ability to detect microbiome associations with disease
Source: PLoS Comput Biol. 2020 Dec 14;16(12):e1008473. doi: 10.1371/journal.pcbi.1008473 (PMC7769610; doi:10.1371/journal.pcbi.1008473)
Supplement: Attachment — Submitted filename: Reviewer-response_v4.docx [file pcbi.1008473.s004.docx]

May 31, 2020

Jason Papin, Ph.D.

Editor-in-Chief, *PLOS Computational Biology*

Dear Dr. Papin,

Thank you for considering a revised version of our manuscript, “A joint modeling approach for longitudinal microbiome data improves ability to detect microbiome associations with disease.” We appreciate the time spent by the editors and the reviewers, and we have responded to all the comments in the responses below. We believe the revised version of the manuscript is much improved because of the reviewer and editor comments.

Please let me know if you have any further questions or comments. We look forward to your review.

Sincerely,

Chad A. Shaw

(on behalf of the authors)

­­­­­­­______________________________________________________________________________

**Comments to the Authors:
Please note here if the review is uploaded as an attachment.**

**Reviewer #1: The authors developed a joint modeling approach that combines longitudinal microbiome reads data and time-to-event outcomes into a single framework. Results show that the model predictions improve the ones from the limited literature, even thought this comparison might not be completely fair, and not all the models are able to incorporate all data available. The article is very well written with a sound statistical formulation.**

We thank the reviewer for their kind words. **Minor concerns:
- In the simulation study, 1000 subjects were used. This would restrict the validity of the conclusions to just the most ambitions projects, since most studies don't have 1000 subjects. It would be interesting to see how the model fairs with fewer data.**

We thank the reviewer for highlighting the importance of the applicability of our methodology to real data sets. We have examined the sample size restrictions of our approach, both related to the number of subjects observed (N) and the number of longitudinal samples collected (K), by comparing error rates of the predicted taxon abundance effect size for varying N and K. Our results demonstrate that for smaller sample sizes (N and K) our method remains quite powerful. Specifically, we found that effect size predictions remained highly accurate with sample sizes as small as N=100 with any number of longitudinal samples. While the effect size predictions with sample size N=50 were slightly less accurate, the performance is improved with increased number of longitudinal samples K.

To clarify these points, we have included a supplementary figure to illustrate these performance metrics and outlined this analysis in the text on page 9, lines 294-303:

“To understand how this methodology performs on smaller datasets, we examined how the number of subjects (N) and number of longitudinal samples (K) affect the model’s accuracy in estimating the taxon abundance effect size. For each combination of N=50,100,100 and K=3,5,10, we simulated 100 microbiome joint model data sets using randomly selected parameter values consistently across all combinations. We compared the taxon abundance effect sizes estimated using our methodology to the true parameter values. The results for this performance analysis, shown in S1 Fig, illustrate that the error rates for parameter estimates did not increase dramatically with fewer subjects or longitudinal samples.”

**S1 Fig. Taxon abundance effect size errors using varying sample sizes.** Application of the joint modeling methodology on simulated data sets with varying sizes for the number of subjects (N) and number of longitudinal samples (K) shows that the model retains accuracy with smaller sample sizes. The mean squared errors (MSEs) for the effect size predictions remain low with sample sizes as small as N=100 with any number of longitudinal samples. The MSEs for effect size predictions are larger with sample size N=50, but the MSEs are reduced with an increased number of longitudinal samples K.

**- The authors claim that since relative abundances often do not follow a Gaussian distribution, a modelling using a negative binomial distribution would be more appropriate, but as they also noted not all taxa follow this distribution. Some explanation of which assumption is closer to reality would help for clarity.**

We have clarified this point on page 11, lines 391-399 by stating:

“Another limitation of our model is the restriction of the longitudinal submodel to a negative binomial distribution, which precludes the analysis of taxa with bimodal distributions or excess zeroes. The number of taxa with these distributional complications is often low but can be highly dependent on the microbial diversity within the dataset. Taxa with an overabundance of zero counts might be better modeled using zero-inflated or hurdle models, but these solutions not currently implemented in existing joint model software. In these situations our negative binomial approach could still be applied but with a reduction in statistical power and performance due to lack of model fit.”

**- I worry about the applicability of the method since it is not well suited for modelling more than a few taxa at a time.**

This is an understandable concern and one we have spent considerable time contemplating. In the context of translational research, we have found that biologists are often interested in identifying the strongest effects that are most easily interpretable. While higher order effects (e.g., multi-taxa correlation effects) are of interest, these complex relationships are commonly still observable in the analyses of individual taxa. That is, strong multi-taxa effects are often detectable by marginal analysis of individual taxa. Moreover, post-processing of the marginal results on individual taxa can reveal these higher order relationships. In the situation of weak effects, single taxon analysis could have low sensitivity, and multi-taxa modeling could improve statistical power. This gap in statistical power is a valid opportunity for statistical research. However, we argue that for biomedical researchers, the circumstance of weak high order effects would not be compelling opportunities for intervention or biomarker discovery.

As we point out, our software has multi-taxa capability for up to three taxa. Despite this limited multi-taxa capability, we would still encourage users to focus on the individual taxon analyses and results. To overcome the limitation of a single taxon analysis, we suggest users perform parallel analyses on multiple potentially relevant taxa. The joint modeling software uses a hierarchical Bayesian approach that does not have the same inherent multiple comparison issues as frequentist methods, so it would not be necessary to correct for multiple testing. More specifically, the model uses zero-centered Gaussian prior distributions for the covariates, which provides model estimates that are inherently more conservative since their posterior distributions are more likely to include zero^[1]^. We have updated the discussion to address these concerns on page 11, lines 364-390:

“Although our novel methodology solves a problem not previously addressed in the field of microbiome research, there remain opportunities for future research in this area. In its current implementation, our model examines the relationship of an individual microbiome taxon and additional covariates with a time-to-event outcome. This

approach can be applied in parallel across individual taxa to perform a comprehensive analysis. We recommend using this parallel analysis approach on a methodically selected subset of individual taxa. The Bayesian hierarchical modeling approach utilized in the joint modeling software produces conservative model estimates that obviate the need for multiple testing corrections. An alternative approach is to model the combined longitudinal dynamics and correlations of many taxa at once within the joint modeling framework. Although of interest, the actualization of joint modeling for many taxa is difficult due to the computational complexity of hierarchical Bayesian analyses,

where the model complexity grows exponentially in the number of parameters considered. The current implementation of the joint model provides functionality for a multivariate joint model that could model up to three taxa; however, this implementation could violate model assumptions due to the dependency issues intrinsic

to microbiome and compositional data. In the future high dimensional Bayesian approaches may enable such model estimation.

We argue that the single taxon joint analysis is effective for two reasons. First, we note that clinicians and biologists focus their interest on the largest and most easily interpretable effects – such as the risk impact of individual taxa on outcomes that we demonstrated in the preterm birth application. Higher order effects of many taxa are less interpretable and therefore less actionable. Second, we note that commonly used analytic methods that consider the entire microbial community by clustering data often result in microbiome profiles dominated by a single taxon. In these instances, a cluster that is driven by an individual taxon is used in downstream association analyses, which is analogous to our approach.”

**Reviewer #2: This paper presents an approach for identifying disease-associated microbiota members in longitudinal data. My expertise is in software engineering for microbiome research so will provide comments on that topic.

While the modeling approach may be novel (not my expertise) I don't expect to see a lot of use of this approach by the microbiome research community.

First, as the authors note, the model will only be relevant to taxa whose abundance profile follows a negative binomial distribution. The authors advise performing analysis external to their software to determine if the software is applicable to a specific taxon. Most users will either ignore this warning, or choose to use another software package. I recommend that the authors include the test that they feel users should apply as part of their software to determine if assumptions of their approach are violated.**

In this manuscript, we present a methodology for performing a novel type of longitudinal microbiome analysis and provide software that extends an existing R package for joint modeling. Because we intend to merge our changes into the existing software and testing for goodness-of-fit for a distribution is outside the scope of that software, we have not introduced code to perform these simple tests. To improve the usability of our software, we have constructed a thorough vignette/tutorial that demonstrates the application of our software to real data and discusses the statistical methodology behind our implementation. We have included examples and instructions for assessing the fit of taxa distributions in the tutorial which we have added as a supplement to the manuscript, referenced on page 5, lines 147-148.

**Next, as far as I can tell, there is not a release version of the software, and there is insufficient description of how to apply this approach. I recommend that the authors create an official release of the software, and provide a tutorial illustrating how it can be applied to example data available in a standard file format (e.g., the biom format, which is widely used by microbiome software tools). Without this, microbiome researchers will not know how to apply this method.**

We appreciate the request for usable software with easily comprehensible documentation. We have updated the text to point to a publicly available version of the software that can be used to implement this methodology. These changes can be found in the “Software implementation” subsection on page 5, lines 143-148.

“We have extended the joint modeling rstanarm software (available on GitHub at https://github.com/pamelanluna/rstanarm) to include the functionality necessary to apply this methodology. Code for replicating our analyses is included in supplemental file S1 Code. Furthermore, we have included a tutorial for preprocessing and analyzing data using our methodology in supplemental file S1 Appendix.”

We have also introduced a supplemental tutorial in this resubmission with detailed instructions on how utilize our software with standard OTU, taxonomy, and metadata tables. The data is imported using the phyloseq R package that includes functionality to import data from other standard formats (e.g. biom file format) which we mention in the tutorial.

**If the above comments are addressed, I'll better be able to evaluate this software and provide a prospective as a potential user of the approach.

Finally, it sounds like the user really has to know specifically what microbes they're interested in before this method can be applied (between 1 and 3 community members). It is extremely uncommon for users to have this information in advance in practice.**

We appreciate the reviewer’s comment from a software engineering perspective. Our work is a new statistical methodology for longitudinal microbiome data – it is intended for use by statisticians and bioinformaticians with the skills to perform the appropriate preliminary analyses to determine potentially relevant taxa. Our resubmission introduces a comprehensive vignette demonstrating application of our methodology. We have included suggestions for possible exploratory analyses to analytically determine taxa to examine using our approach in this new supplemental tutorial, referenced on page 5, lines 147-148.

**Line 236: I disagree with this assertion - just because this is what may have been identified in the past, it doesn't mean that interactions between community members are less important.**

The authors agree that interactions between community members are important to understanding the dynamics of the microbiome. In the referenced statement, we aim to assert that some common analytic methods that consider the entire community still result in models that truly only examine the abundances of a few taxa. In those instances, our methodology would be comparable. We have clarified this point on page 11, lines 386-390 by stating:

“Second, we note that commonly used analytic methods that consider the entire microbial community by clustering data often result in microbiome profiles dominated by a single taxon. In these instances, a cluster that is driven by an individual taxon is used in downstream association analyses, which is analogous to our approach.”

**Reviewer #3: Luna et al. proposed a joint modeling approach for longitudinal microbiome data with survival outcome to detect microbiome associations with disease. The proposed method uses a negative binomial mixed effects model jointing with a Cox hazard model in longitudinal setting with the capability to measure how much changes in the microbiome affect disease onset(survival outcome).

This is very challenge topic in microbiome literature given that the microbiome data are very complicated: structured with tree, compositional, multivariate, sparse and often with many zeros.
Due to the challenge, the development of longitudinal and survival model for analyzing microbiome data are rare. So far only several longitudinal models and two survival models have been proposed. Thus, the proposed method will add a statistical tool to the literature of microbiome methods and provide an alternative to model longitudinal and survival microbiome data. However, the proposed method also has several limitations.**

We thank the reviewer for acknowledging the complexity of this problem and the novelty of our proposed solution. **1. The model analyzes one taxon at a time. Actually, the univariate approach cannot account for the compositional nature of microbiome data as claimed by the authors because this approach treats each of taxon as independent and does not consider each taxon in ecosystem.**

We use the phrase “compositional nature” to convey that in the context of the event submodel we are looking at the proportion of the taxon abundance relative to the total number of reads in the sample rather than the raw count values. To clarify that we are not considering the entire community, we have updated the text to use the term “proportional” rather than “compositional” where appropriate.

**2. To join the longitudinal negative binomial mixed effects model to survival model, the proposed method introduces “a parameterization that represents the estimated longitudinal submodel values as scaled relative abundances in the event submodel to address the compositional nature of microbiome data and to improve model interpretability.”

Since the proposed method did not use any log-ratio transformation, only scaling the abundant counts into relative abundances could not address the compositional nature of microbiome data.**

We state that we “address the compositional nature of microbiome data” to highlight the important distinction between the use of count values in the longitudinal submodel and relative abundances in the event submodel. The inclusion of count values in the event submodel would give varying weight to samples with differing numbers of total reads, so it is imperative that the count values are transformed into relative abundances for an equitable representation across samples. We have updated this statement on page 3, lines 72-75 to read:

“We additionally introduce a parameterization that represents the estimated longitudinal submodel values as scaled relative abundances in the event submodel to address the proportional nature of microbiome data and to improve model interpretability.”

**Actually in the simulation study, the relative abundances are assumed to be distributed as normal. Authors need to clarify this confusion.**

In the simulation study, we assume that the taxon abundance count values follow a negative binomial distribution (page 5, line 158) and that the total number of reads in a sample follows a normal distribution (page 5, lines 166-167). We do not place any direct assumptions on the distribution of the relative abundances, and it would be unnecessary to do so since they are independent variables in the event submodel.

**3. Generally the univariate approach is underpowered compared to multivariate approach and ignores the large p and small n problem. Can the authors comment on how to detect the association between many taxa (e.g., 1000 taxa) and disease onset using the proposed method? Is to test one by one and then adjust for multiple testing?**

Although there is a loss of power with a univariate approach versus a multivariate approach, our model benefits from increased power due to the inclusion of longitudinal microbiome data in the model. Moreover, we have found that biologists are often interested in identifying the strongest effects that are most easily interpretable and that multi-taxa high order effects are difficult for them to interpret. To overcome the limitation of a single taxon in the univariate model, we suggest users perform multiple testing on multiple potentially relevant taxa. The joint modeling software uses a hierarchical Bayesian approach that does not have the same inherent multiple comparison issues as frequentist methods, so it would not be necessary to correct for multiple testing. More specifically, the model uses zero-centered Gaussian prior distributions for the covariates, which provides model estimates that are inherently more conservative since their posterior distributions are more likely to include zero^[2]^. We have updated the discussion to address these questions on page 11, lines 364-390:

“Although our novel methodology solves a problem not previously addressed in the field of microbiome research, there remain opportunities for future research in this area. In its current implementation, our model examines the relationship of an individual microbiome taxon and additional covariates with a time-to-event outcome. This

approach can be applied in parallel across individual taxa to perform a comprehensive analysis. We recommend using this parallel analysis approach on a methodically selected subset of individual taxa. The Bayesian hierarchical modeling approach utilized in the joint modeling software produces conservative model estimates that obviate the need for multiple testing corrections. An alternative approach is to model the combined longitudinal dynamics and correlations of many taxa at once within the joint modeling framework. Although of interest, the actualization of joint modeling for many taxa is difficult due to the computational complexity of hierarchical Bayesian analyses,

where the model complexity grows exponentially in the number of parameters considered. The current implementation of the joint model provides functionality for a multivariate joint model that could model up to three taxa; however, this implementation could violate model assumptions due to the dependency issues intrinsic

to microbiome and compositional data. In the future high dimensional Bayesian approaches may enable such model estimation.

We argue that the single taxon joint analysis is effective for two reasons. First, we note that clinicians and biologists focus their interest on the largest and most easily interpretable effects – such as the risk impact of individual taxa on outcomes that we demonstrated in the preterm birth application. Higher order effects of many taxa are less interpretable and therefore less actionable. Second, we note that commonly used analytic methods that consider the entire microbial community by clustering data often result in microbiome profiles dominated by a single taxon. In these instances, a cluster that is driven by an individual taxon is used in downstream association analyses, which is analogous to our approach.”

We would also contend that it is unrealistic to expect to analyze such a large magnitude of taxa. In order to have a detectable effect on the time-to-event, a taxon would need to be present in a substantial number of samples. This will unlikely be the case for 1000, or even 100, taxa. To reduce the number of univariate models the user will need to construct, we have included in the new supplemental tutorial suggestions for possible exploratory analyses to analytically determine potentially relevant taxa to examine using our methodology.

**4. Since the proposed method uses a negative binomial mixed effects model to determine longitudinal taxon abundances, it is better to provide a procedure to detect the taxa distribution for NB model for guiding the users to appropriately use the method.**

In this manuscript, we present a methodology for performing a novel type of longitudinal microbiome analysis and provide software that extends an existing R package for joint modeling. To improve the usability of our software, we have constructed a thorough vignette/tutorial that demonstrates the application of our software to real data and discusses the statistical methodology behind our implementation. We have included examples and instructions for assessing the fit of taxa distributions in the tutorial which we have added as a supplement to the manuscript, referenced on page 5, lines 147-148.

**5. Since this is methodology paper, the methods are more important, so the Methods Part should be followed by the Introduction part for better presentation.**

We appreciate the reviewer’s suggestion for improving the presentation of our paper. We have updated our manuscript so that the Methods section follows the Introduction section.


**Minor:

The authors stated that “One method for determining associations between microbial compositions and event times has been developed [24],…”

Actually, we just reviewed, except for reference #24, there has another model for microbiome-based association test for survival outcomes.

Koh, H., A. E. Livanos, M. J. Blaser and H. Li (2018). "A highly adaptive microbiome-based association test for survival traits." BMC genomics 19(1): 210-210.**

We thank the reviewer for directing us to this article. We have included a reference to the publication in our manuscript and updated the text accordingly on page 2, lines 20-23.

“Two methods for determining associations between microbial compositions and event times have been developed, but they only examine the microbiome composition at a single time point.”

1. Gelman, A., Hill, J., & Yajima, M. (2012). Why We (Usually) Don’t Have to Worry About Multiple Comparisons. *Journal of Research on Educational Effectiveness*, *5*(2), 189–211. https://doi.org/10.1080/19345747.2011.618213
2. Gelman, A., Hill, J., & Yajima, M. (2012). Why We (Usually) Don’t Have to Worry About Multiple Comparisons. *Journal of Research on Educational Effectiveness*, *5*(2), 189–211. https://doi.org/10.1080/19345747.2011.618213
