# Supplementary material for: A joint modeling approach for longitudinal microbiome data improves ability to detect microbiome associations with disease
Source: PLoS Comput Biol. 2020 Dec 14;16(12):e1008473. doi: 10.1371/journal.pcbi.1008473 (PMC7769610; doi:10.1371/journal.pcbi.1008473)
Supplement: Attachment — Submitted filename: mbjm-reviewer-response-2_v3.docx [file pcbi.1008473.s005.docx]

September 12, 2020

Jason Papin, Ph.D.

Editor-in-Chief, *PLOS Computational Biology*

Dear Dr. Papin,

Thank you for considering a revised version of our manuscript, “A joint modeling approach for longitudinal microbiome data improves ability to detect microbiome associations with disease.” We appreciate the time spent by the editors and the reviewers and have responded to the remaining comments in the responses below. We believe the revised version of the manuscript is much improved thanks to reviewer feedback.

We would like to stress that our manuscript is a methodologic contribution that addresses an important gap in statistical science for microbiome analysis and has possible extensions to other omics data. We have shown that our method can be used to provide novel biological insights through its application to a microbiome study dataset presented in our work. As part of this manuscript, we also introduce a software contribution that facilitates the accessibility of our methodology and we include a comprehensive vignette demonstrating its use.

Please let us know if you have any further questions or comments. We look forward to your review.

Sincerely,

Chad A. Shaw

(on behalf of the authors)

­­­­­­­______________________________________________________________________________

**Reviewers’ comments to the Authors:**

**Reviewer #1:**

**All my concerns have been addressed**

**Reviewer #2:**

**Again, my assessment is that the new methodology sounds interesting but I have serious concerns about the software.**

**The authors have partially addressed my concerns about usability of the software through the development of their vignette.**

**Comment 1: I strongly recommend that the authors make their vignette accessible through their website and setup some kind of continuous integration testing to ensure that the vignette does not become outdated (this would alert them for example if a code change resulted in their vignette not working anymore). As anyone working in bioinformatics knows, it's both common and frustrating to encounter tutorials that are out of date with respect to software. Continuous integration testing of tutorials is really the only effective way to ensure that this doesn't happen.**

Response: We appreciate the feedback and points by the reviewer. We have made the vignette publicly available via GitHub at <https://pamelanluna.github.io/mbjm-tutorial/>. We have updated the paper on page 5, lines 148-151 to include a link to the web version of our vignette: “Furthermore, a tutorial for preprocessing and analyzing data using our methodology is available online at <https://pamelanluna.github.io/mbjm-tutorial/> and is included here as S1 Appendix.”

We understand the reviewer’s frustration with outdated tutorials, and their suggestion regarding continuous integration testing is on-point from the perspective of software development. However, we emphasize that our paper is a methodologic contribution and a signficant innovation in statistical science for time-to-event analysis with microbiome data, with potential applications for omics data more generally. While the software and vignette are important for demonstrating the efficacy of our model and providing readily available tools for its application, they are not the primary focus of this work. Integration testing for the rstanarm software is maintained by the developers and has been completed for our contributions provided to the rstanarm team. Our vignette is a comprehensive guide to using our new methodology with the software implementation we have made available. We will update the software installation instructions included in this vignette once our changes to the rstanarm package are released on CRAN.

**Comment 2: The repository that this code was forked from now has >60 commits since the authors' initial fork and there is currently a merge conflict that will prevent a merge upstream without first being addressed (not sure how serious the conflict is and whether it impacts the authors' functionality). Will the authors' code be kept up-to-date with the original source? It's possible that one or more of those commits included essential bug fixes which won't be available to your users.**

Response: We agree with the reviewer’s assessment of the difficulty of maintaining the concordance of our code changes with the upstream code. The merge conflicts have been resolved. Our updates have now been approved by the rstanarm authors and are being integrated into the development version of rstanarm.

**Comment 3:**  **The typical practice for extending a well-maintained software package like rstanarm would be to submit your improvements upstream to the original codebase rather than fork the code and try to maintain a parallel fork. If you're not already in touch with the developers of rstan, just as a word of warning, they might not appreciate your approach. I didn't notice that this was what you were doing on my first review. Forking a project and going your own direction with it is generally considered rude, even though open source licenses (GPL in this case) technically allow it. This is because it can create more work for the original development team. (I apologize if I'm misunderstanding and the authors of this paper are also part of the team developing rstanarm - I may be misunderstanding what is happening here.) It seems that the rstanarm authors have contribution guidelines:** [**http://mc-stan.org/rstanarm/dev-notes/index.html**](http://mc-stan.org/rstanarm/dev-notes/index.html)

Response: We acknowledge these thoughtful comments by the reviewer regarding conventions and etiquette in software development. As would be appropriate, we strictly followed the contribution guidelines for rstanarm referenced above as well as the developer guidelines for the core Stan software: <https://mc-stan.org/developers/>. We contacted the rstanarm developers prior to making any changes to ensure that our modifications would be appropriate for the software. The developers have been extremely supportive of these additions, which introduce functionalities that are practicable outside the scope of this paper, and are appreciative of our contributions to the software package.

**Comment 4: As a result of the authors’ approach of forking a repository but not pushing changes back upstream to the source, the README.md file on their GitHub repository actually doesn’t show how to install their software, but rather the upstream software that doesn’t contain their changes:**
[**https://github.com/pamelanluna/rstanarm/blob/master/README.md**](https://github.com/pamelanluna/rstanarm/blob/master/README.md) **You’re also effectively telling your users to go to the original developers for technical support based on that README file. Again, this is something they might not appreciate (and again, I apologize if I’m misunderstanding something here), especially if they’re not supportive of the modifications you made.

The authors mention that their goal is to submit the code back upstream in their response to reviewers (“Because we intend to merge our changes into the existing software”…"). In my view this is essential if you want to include anything about this software in the publication.**

Response: We thank the reviewer for sharing their concerns about the accessibility of the software. Our contributions to the rstanarm software package have been approved by the authors. The changes are being merged into the development version of rstanarm and will be included in the next release. We have updated the software implementation section of the paper on page 5, lines 142-147 to direct users to the rstanarm GitHub repository:

“This methodology can be implemented using the rstanarm R package, which provides tools for Bayesian statistical inference of applied regression models, including the joint model for longitudinal and time-to-event data [41, 42]. We have extended the joint modeling rstanarm software to provide the functionality necessary to apply this approach (currently available via the development version of rstanarm on GitHub at https://github.com/stan-dev/rstanarm).”

**Reviewer #3:**

**The authors have answered all my questions. The reversion has been significantly improved including presentation, clearity, and discussion of its limitations.**
